# Identification of *Quercus agrifolia* (coast live oak) resistant to the invasive pathogen *Phytophthora ramorum* in native stands using Fourier-transform infrared (FT-IR) spectroscopy

**DOI:** 10.3389/fpls.2014.00521

**Published:** 2014-10-14

**Authors:** Anna O. Conrad, Luis E. Rodriguez-Saona, Brice A. McPherson, David L. Wood, Pierluigi Bonello

**Affiliations:** ^1^Department of Plant Pathology, The Ohio State UniversityColumbus, OH, USA; ^2^Department of Food Science and Technology, The Ohio State UniversityColumbus, OH, USA; ^3^Department of Environmental Science, Policy, and Management, University of CaliforniaBerkeley, CA, USA

**Keywords:** coast live oak, resistance, sudden oak death, infrared spectroscopy, predictive modeling

## Abstract

Over the last two decades coast live oak (CLO) dominance in many California coastal ecosystems has been threatened by the alien invasive pathogen *Phytophthora ramorum*, the causal agent of sudden oak death. In spite of high infection and mortality rates in some areas, the presence of apparently resistant trees has been observed, including trees that become infected but recover over time. However, identifying resistant trees based on recovery alone can take many years. The objective of this study was to determine if Fourier-transform infrared (FT-IR) spectroscopy, a chemical fingerprinting technique, can be used to identify CLO resistant to *P. ramorum* prior to infection. Soft independent modeling of class analogy identified spectral regions that differed between resistant and susceptible trees. Regions most useful for discrimination were associated with carbonyl group vibrations. Additionally, concentrations of two putative phenolic biomarkers of resistance were predicted using partial least squares regression; >99% of the variation was explained by this analysis. This study demonstrates that chemical fingerprinting can be used to identify resistance in a natural population of forest trees prior to infection with a pathogen. FT-IR spectroscopy may be a useful approach for managing forests impacted by sudden oak death, as well as in other situations where emerging or existing forest pests and diseases are of concern.

## Introduction

Sudden oak death is a highly destructive disease that has caused extensive mortality of oaks and tanoaks in coastal central and northern California and southwest Oregon over the last two decades (Rizzo et al., 2002; reviewed in Rizzo and Garbelotto, 2003; McPherson et al., 2005, 2010; Meentemeyer et al., 2008; Brown and Allen-Diaz, 2009; Davis et al., 2010; Cobb et al., 2012). Few management options exist for controlling the disease and they are all centered on preventative management and silvicultural practices (reviewed in Rizzo and Garbelotto, 2003) since little, if anything, can be done once trees become infected (reviewed in Grunwald et al., 2008). Individual high value trees can be protected by treating them preventatively with phosphonate-based fungicides (Garbelotto and Schmidt, 2009). However, arguably the best management practice for sudden oak death in oak woodlands would focus on the identification and utilization of resistant germplasm, since genetic resistance is the cornerstone of plant protection against insect pests and diseases in conducive environments.

Coast live oak (CLO—*Quercus agrifolia* Née) is a highly susceptible host of *Phytophthora ramorum* Werres et al., the causal agent of sudden oak death. During the early to mid-2000s, the CLO infection rate in some populations was found to be as high as 5.0% y^−1^, with a mortality rate of 3.1% y^−1^ (McPherson et al., 2010). In sites heavily impacted by the disease, the loss of CLO basal area over a 20 year period was predicted to be 59–70% (Brown and Allen-Diaz, 2009). Even with high infection and mortality rates, variation in CLO susceptibility to the pathogen has been observed in laboratory assays (Dodd et al., 2005) and within natural populations in field studies (McPherson et al., 2005; Ockels et al., 2007; Nagle et al., 2011). Trees considered naturally resistant to *P. ramorum* never show symptoms of infection (e.g., bleeding exudate and discoloration of phloem tissue), they do not host bark and ambrosia beetles—often associated with infection (Rizzo and Garbelotto, 2003; McPherson et al., 2005, 2008), or appear to recover following infection (Nagle et al., 2011; McPherson et al., 2014). Additionally, when trees are artificially inoculated with a pathogen, resistance can be defined based on canker length, where resistant trees are those with canker lengths that do not differ significantly from mock inoculations or with canker lengths below some critical threshold—a criterion that has been used for pine (Gordon et al., 1998) and more specifically for CLO infected with *P. ramorum* (McPherson et al., 2014).

While the mechanism(s) of CLO resistance to *P. ramorum* is unknown, some studies support the hypothesis that plant specialized metabolites, in particular phenolic compounds, are important for CLO defense against *P. ramorum* (Ockels et al., 2007; Nagle et al., 2011). Moreover, Stong et al. (2013) found that tannin-enriched extracts and ground foliage from Oregon white oak (*Quercus garryana* Dougl. Ex Hook) and California black oak (*Q. kelloggii* Newberry), a susceptible host of *P. ramorum*, adversely affect the growth of *P. ramorum*. Tannin-enriched extracts also inhibited the production of *P. ramorum* zoospores and elicitin, which is positively correlated with *P. ramorum* growth and zoospore production (Stong et al., 2013). A reanalysis of Nagle et al. (2011) phenolic data (McPherson et al., 2014) revealed that concentrations of certain phenolic compounds (hereafter referred to as putative phenolic biomarkers of resistance), quantified from asymptomatic phloem of trees already infected with *P. ramorum*, could be used to identify resistant CLO. One of these biomarkers is ellagic acid, a byproduct of ellagitannin hydrolysis (Ascacio-Valdes et al., 2011), which has previously been associated with CLO defense and resistance (Ockels et al., 2007; Nagle et al., 2011). Ellagic acid has also been shown to inhibit the growth of certain invasive *Phytophthora* species *in vitro*, including *P. cinnamomi* Rands (Cahill and McComb, 1992), a generalist pathogen associated with oak decline throughout North America and Europe (Brasier, 1996; Tainter et al., 2000; Nagle et al., 2010) and *P. ramorum* (McPherson et al., 2014), in the latter case at *in planta*-relevant concentrations. Still, CLO resistance cannot be predicted by measuring the concentration of a single phenolic compound, but instead can be predicted only when several phenolic compounds are used concurrently in a predictive model (McPherson et al., 2014). Thus, techniques that examine a broader spectrum of plant-derived chemicals may be more useful for the identification of resistant CLO.

One technique that is capable of producing comprehensive chemical fingerprints is Fourier-transform infrared (FT-IR) spectroscopy. FT-IR spectroscopy has many advantages over more traditional methods of chemical fingerprinting (e.g., high performance liquid chromatography-mass spectrometry), such as its rapidity and reproducibility in the analysis of solid, liquid, or gaseous samples (Fiehn, 2001). Infrared (IR) spectroscopy can be used to produce chemical fingerprints, which then can be used to identify or discriminate between samples, because spectra, which are produced by measuring changes in the molecular absorption of IR radiation, are determined based on qualitative and quantitative attributes of the chemicals (i.e., functional groups) present in a given sample (Diem, 1993; Guillén and Cabo, 1997; reviewed in Rodriguez-Saona and Allendorf, 2011). This is because the molecular structure of compounds influences how IR radiation is absorbed and consequently the mechanical motion of the molecules (either vibrational or rotational) (Diem, 1993; Guillén and Cabo, 1997; reviewed in Rodriguez-Saona and Allendorf, 2011).

While spectroscopy has been used to determine the water status of CLO foliage (Hunt and Rock, 1989; Pu et al., 2003; Cheng et al., 2011) there are no reports of its use for chemically fingerprinting CLO phloem tissue. However, FT-IR spectroscopy has been used to monitor specialized metabolite production in grapevine (Schmidtke et al., 2012) and for qualitative and semi-quantitative analysis of birch bark extracts (Cîntă-Pînzaru et al., 2012). The technique was also used successfully to identify markers of potato resistance to late blight disease caused by *Phytophthora infestans* (Mont.) de Bary (Taoutaou et al., 2012), to monitor chemical changes in elm wood following infection with the pathogen *Ophiostoma novo-ulmi* Brasier (a causal agent of Dutch elm disease) (Martín et al., 2005), to distinguish between resistant and susceptible elms post-inoculation (Martín et al., 2005), and was able to discriminate between elm clones of differing levels of susceptibility to *O. novo-ulmi* based on the analysis of healthy tissue (Martin et al., 2008).

Based on the evidence that quantitative differences in the constitutive chemical composition of CLO phloem tissue are associated with resistance to *P. ramorum*, the objectives of this study were to determine if FT-IR spectroscopy could be used to (1) discriminate between resistant and susceptible trees, and (2) predict the concentration of putative phenolic biomarkers of resistance, by analyzing phloem tissue collected prior to infection.

## Materials and methods

### Inoculation and resistance screening

In July 2010, two phloem samples were collected with a cordless drill equipped with a 1.9 cm diameter drill bit from the main stem of 154 haphazardly selected, apparently disease-free (asymptomatic) CLO from two sites (37° 55′ 12.23″ N, 122° 8′ 8.73″ W and 37° 56′ 8.57″ N, 122° 7′ 40.46″ W), covering ~8 ha in total, within Briones Regional Park (Contra Costa Co., CA, USA), an area just outside the then-known area of infestation, for which no records of natural disease incidence were known prior to the start of the study (Brown and Allen-Diaz, 2009) and in which only a small number of symptomatic CLO were observed in the vicinity of the plots at the initiation of the study. Phloem samples from each tree were pooled, placed on dry ice in the field, and then frozen at −18°C until October 2010, when they were shipped on dry ice to The Ohio State University. Following shipping, all samples were stored at −80°C.

In September 2010, each tree stem was inoculated at breast height at the two ends of a 1/3 circumference arc with a plug of *P. ramorum* isolated from an *Umbellularia californica* (Hook. and Arn.) Nutt. (California bay laurel) in Contra Costa Co. and grown on 1/3 V8 medium (inoculum kindly provided by Dr. David Rizzo, UC Davis). Inoculations were performed according to the methods of McPherson et al. (2008). Inoculations were conducted under a California Department of Food and Agriculture permit released to Dr. David Rizzo and were necessary because, at the initiation of this study, no other methods were available to screen trees for resistance.

Resistance was determined by observing trees 10, 14, 22, and 34 months following inoculation and separating them into three groups based on the disease phenotypes of Nagle et al. (2011): *in remission*—trees were initially symptomatic (only bleeding exudate observed) but appeared to recover quickly (no more bleeding)—trees considered most resistant; *symptomatic*—trees continued to bleed and/or beetle activity and/or *Annulohypoxylon* fruiting bodies were observed; and *susceptible*—trees had brown or leafless crowns, or inoculated stems had snapped (some with green foliage)—trees considered dead and most susceptible.

Support for the phenotypic groupings was sought by calculating the mean length of the two external cankers on each tree ~10 months following inoculation (McPherson et al., 2014). Only phloem from trees classified as resistant (i.e., in remission) (*n* = 22) or susceptible (*n* = 24) 22 months post-inoculation (Conrad et al., unpublished) was used for the present analysis, since these trees were considered most resistant and susceptible, respectively, and thus were ideal for testing whether or not FT-IR spectroscopy could be used to distinguish between resistant and susceptible CLO.

### FT-IR spectroscopy

Phloem tissue was finely ground in liquid nitrogen and stored at −80°C. 100 ± 1 mg fresh weight (FW) of finely ground phloem tissue was extracted two times with 0.5 mL of HPLC grade methanol (Fisher Scientific, Pittsburgh, PA, USA) for 24 h at 4°C, as described in Nagle et al. (2011). Extracts were pooled and stored at −80°C until analysis.

The ability of FT-IR spectroscopy and chemometric analysis to discriminate between extracts from resistant and susceptible CLO was then tested on two separate instruments. The first was an Excalibur 3500GX FT-IR spectrometer (benchtop) (Digilab, Randolph, MA, USA), equipped with a triple-bounce zinc selenide, attenuated total reflectance (ATR) accessory and a potassium bromide beamsplitter. The second instrument was a Cary 630 FT-IR spectrometer (portable) (Agilent Technologies Inc., Santa Clara, CA, USA) equipped with a five-bounce zinc selenide ATR accessory. Bounce number indicates the number of times the sample comes in contact with the IR beam. Spectra were collected over a range of 4000–700 cm^−1^ at 4 cm^−1^ resolution and an interferogram of 64 scans was co-added for each sample. Spectral data were displayed in terms of absorbance and viewed using Win-IR Pro Software (Agilent Technologies Inc., Santa Clara, CA, USA).

Methanol extracts were concentrated prior to analysis on the benchtop unit. Methanol was completely evaporated from aliquots of extract using a Savant SpeedVac DNA 120 (Thermo Scientific, Asheville, NC, USA) at room temperature and with a low drying rate. Resulting pellets were re-suspended in methanol to a final concentration of 10 times (10x) that of the original extract. 5μl of 10x extract were loaded onto the ATR accessory crystal and allowed to sit for ~60 s; this allowed methanol, which interferes with the spectra of plant extracts, to evaporate before analysis. For analysis with the portable unit, 50 μl of crude methanol extract were loaded onto the ATR accessory crystal. Samples were brought to dryness using a water aspirator and then analyzed. Two technical replicates were analyzed on each unit for each extract (biological replicate).

### Statistical analysis

#### Confirming resistance phenotypes

Canker length was compared between resistant and susceptible trees using an independent samples *t*-test (IBM SPSS Statistics 21). Assumptions were tested prior to analysis using Shapiro-Wilk's and Levene's tests for normality and homoscedasticity, respectively. Canker length data were log transformed in order to satisfy the assumption of normality and the Welch-Satterthwaite method was used to account for a lack of homoscedasticity between groups (Welch, 1938, 1947; Satterthwaite, 1946; reviewed in Ruxton, 2006).

#### FT-IR spectroscopy

Data collected from the portable unit and benchtop unit were analyzed using the chemometric software Pirouette version 4.0 (Infometrix Inc., Woodville, WA, USA). Soft independent modeling of class analogy (SIMCA) was used to discriminate between resistant and susceptible trees, while partial least squares regression (PLSR) was used to predict the concentration of two putative phenolic biomarkers of resistance, ellagic acid and an uncharacterized flavonoid (FLV1). Ellagic acid and FLV1 were quantified (mg g^−1^ FW) based on HPLC analysis using a modification of the method described in Nagle et al. (2011) and using an ellagic acid standard curve for compound quantification, as described in Ockels et al. (2007) (Conrad et al., unpublished). SIMCA is a classification technique that develops principal components models for each training group (i.e., resistant and susceptible CLO) and identifies variables that are important for discriminating between groups (Subramanian et al., 2007). PLSR uses multivariate analysis to reduce high dimensional, potentially collinear data (e.g., spectral frequencies), and regression analysis to estimate the concentration of variables of interest (e.g., concentrations of phenolic compounds), while maximizing covariance (Wilkerson et al., 2013).

Technical replicates from each biological replicate were analyzed separately. For SIMCA analysis, data were transformed using the standard normal variate (SNV) approach and by taking the second derivative (with a 21 points Savitzky and Golay polynomial filter) (Savitzky and Golay, 1964). For PLSR, data were transformed using the divide by sample 2-norm function. SIMCA 3D class projection plots were used to visualize clustering patterns of resistant and susceptible trees. SIMCA Coomans plots and discriminating power plots were used to identify spectral region(s) that had the highest discriminating power between resistant and susceptible trees (Coomans and Broeckaert, 1986; Subramanian et al., 2007). Coomans plots and 3D class projection plots were also used to identify outliers, which were then removed from the model. For PLSR, loadings and scores plots were used to visualize data and identify the infrared region that best explained the observed variation, respectively. PLSR model performance was evaluated in terms of outlier diagnostics, leave-one-out cross validation, and number of factors included in the model (Wilkerson et al., 2013). Outliers were trimmed based on the methods of Wilkerson et al. (2013), and sample sizes reported reflect the number of technical replicates used for each analysis.

## Results

### Resistant phenotypes

**Figure 1:**
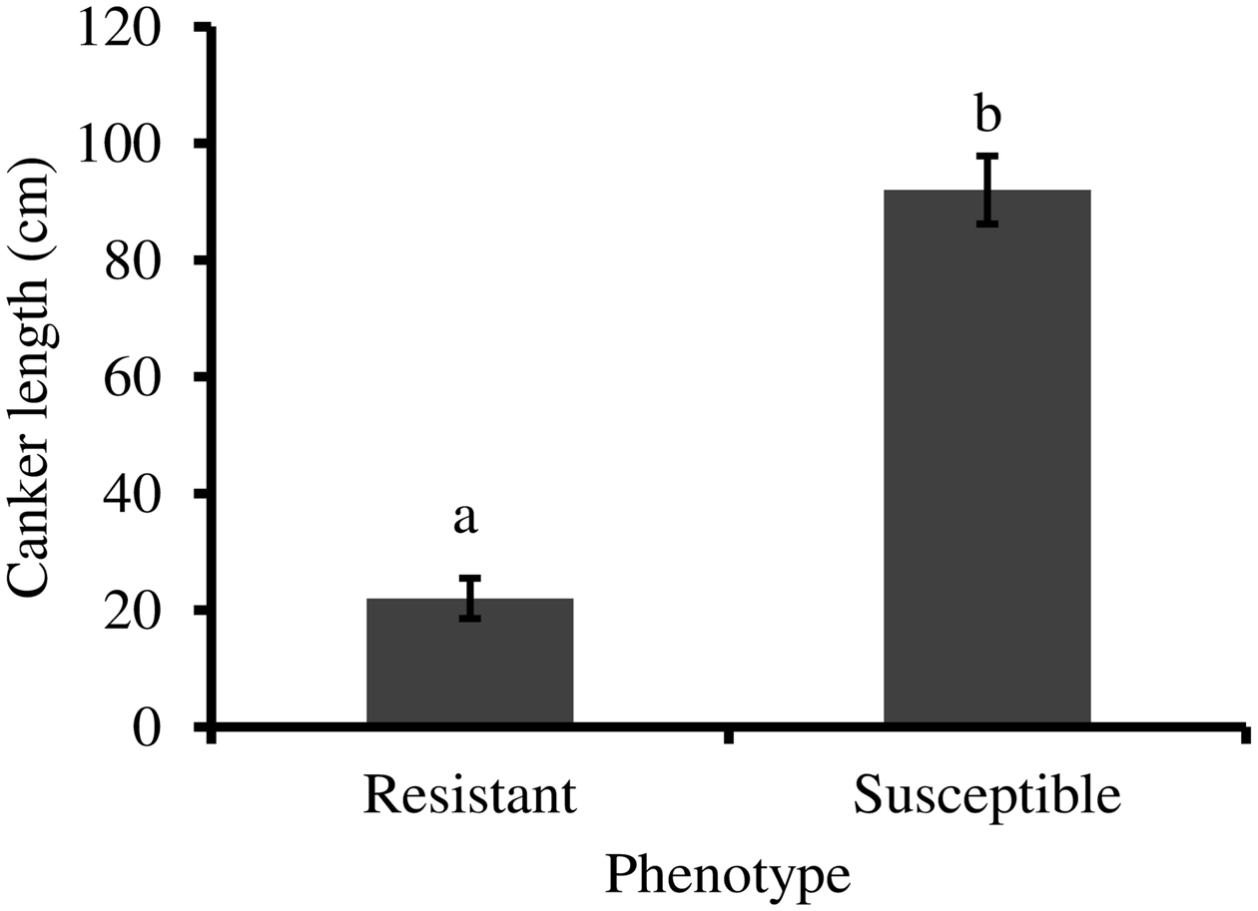
**External canker length (±standard error) measured 10 months following inoculation with *P. ramorum***. Resistant trees (*n* = 22) have significantly smaller canker lengths than susceptible trees (*n* = 24) (independent *t*-test, *P* < 0.001). Statistical analysis performed using log transformed data.

CLO classified as resistant (*n* = 22) in 2012 based on symptom expression had significantly smaller external canker lengths 10 months post-inoculation, compared to trees classified as susceptible (*n* = 24) (*t* = 8.475, *df* = 27, *P* < 0.001) (Figure 1).

### FT-IR spectra and SIMCA analysis

**Figure 2:**
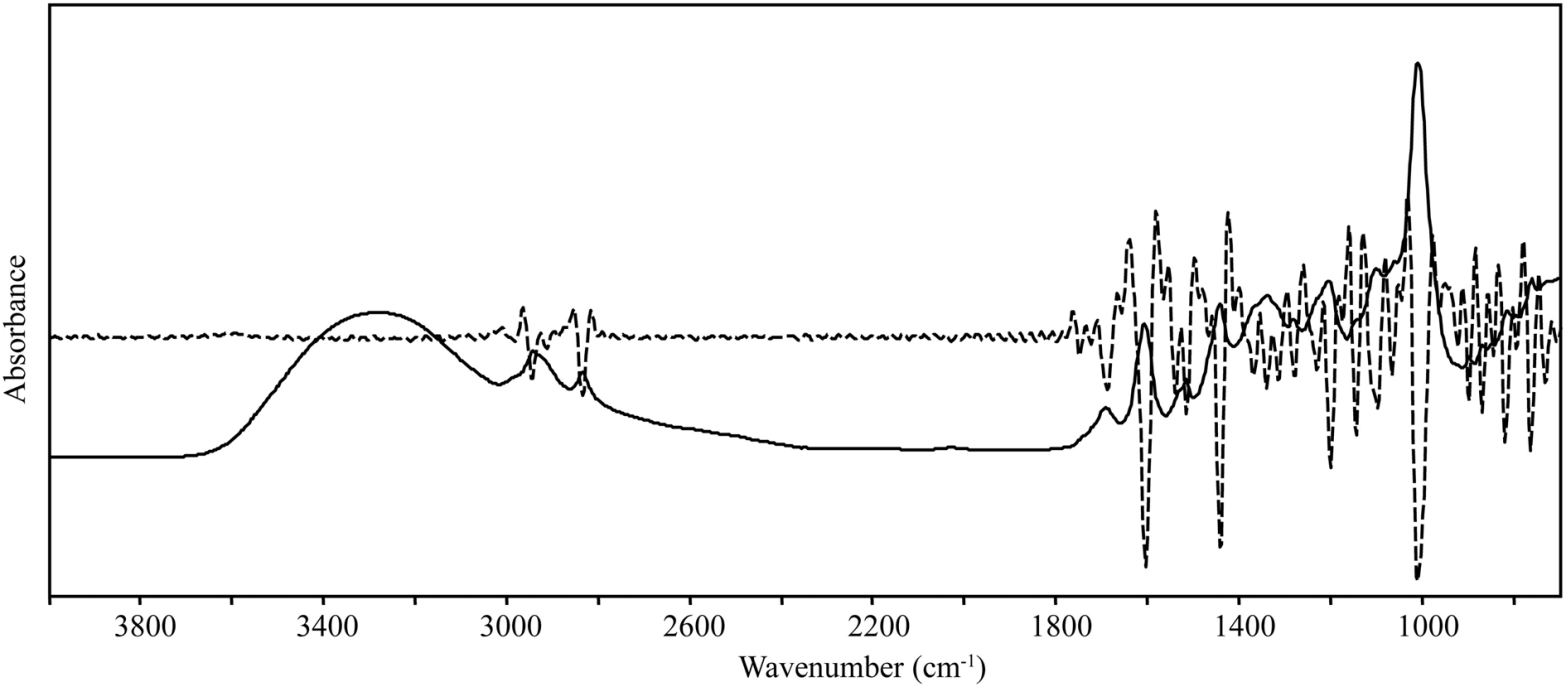
**Solid line—raw infrared spectrum from coast live oak phloem extracts; dashed line—second derivative infrared spectrum**. The second derivative spectrum was used to resolve overlapping bands (peaks) and to identify differences in the spectra of resistant and susceptible trees.

**Figure 3:**
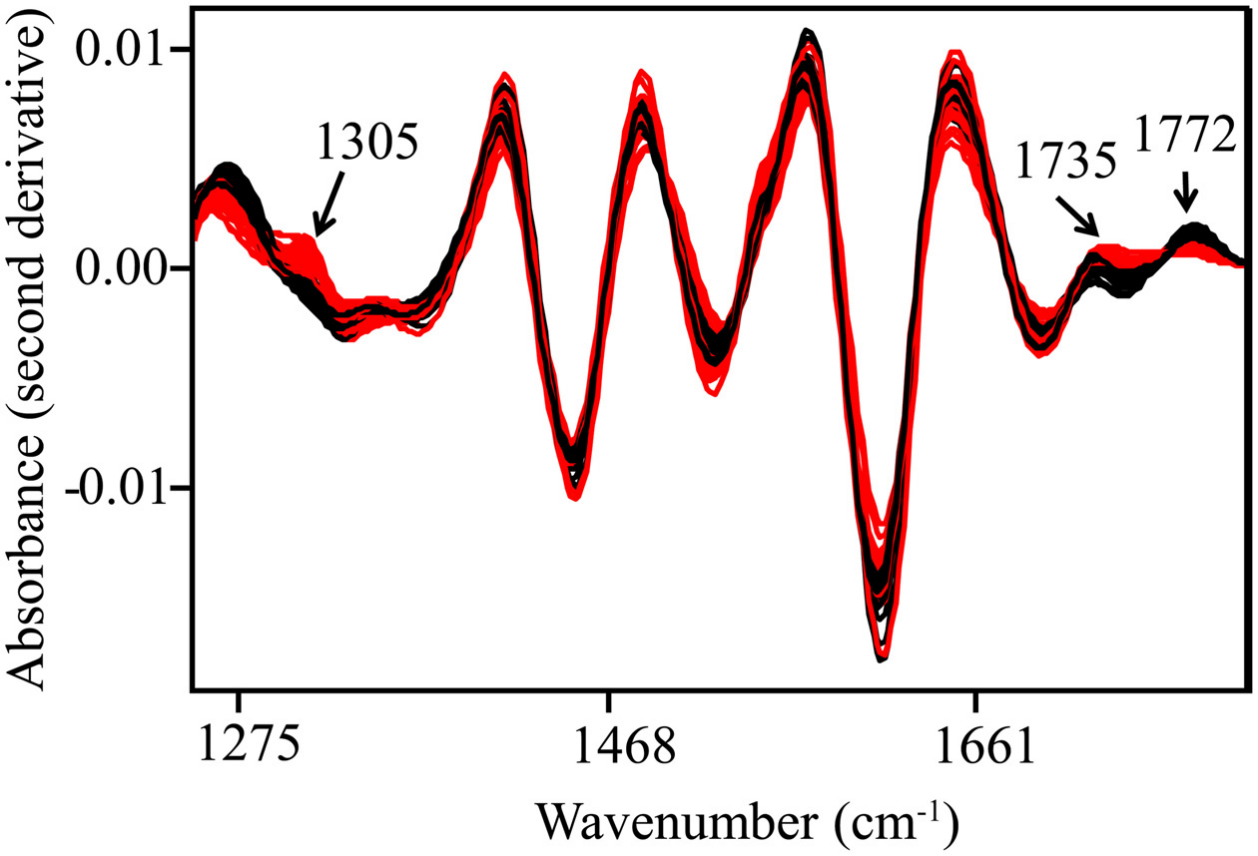
**Second derivative and SNV-transformed spectra**. Spectral bands with high discriminating power are indicated with arrows. Black—resistant trees; Red—susceptible trees.

Spectral data were collected from the mid-IR region (4000–700 cm^−1^), and overlapping peaks were resolved by using SNV and second derivative functions (Figure 2, Table 1). Only quantitative spectral differences were observed between resistant and susceptible trees (Figure 3). Differences between extracts from resistant and susceptible trees were observed only after spectral transformation, and were most visible around 1305, 1735, and 1772 cm^−1^.

**Table 1:** **Wavenumber ranges and associated functional groups**.

**Wavenumber range (cm^−1^)**	**Assignment**	**References**
2840–3040	-C-H (CH_2_) stretching	Diem, 1993; Koca et al., 2010
2860–2760	-C-H (CH_2_) stretching	Diem, 1993; Koca et al., 2010
1650–1740	C=O stretching	Diem, 1993
1520–1650	C=C (benzene ring)	Martín et al., 2005
1400–1480	C-O stretching, CH_2_, CH_3_	Diem, 1993; Guillén and Cabo, 1997
1400–1180	C-O stretching, CH_2_ stretching, C=O	Koca et al., 2010; Carballo-Meilan et al., 2014
1200–800	C-O stretching (carbohydrate region)	Martín et al., 2005

*Spectral ranges based on peak presence in raw infrared spectrum*.

**Figure 4:**
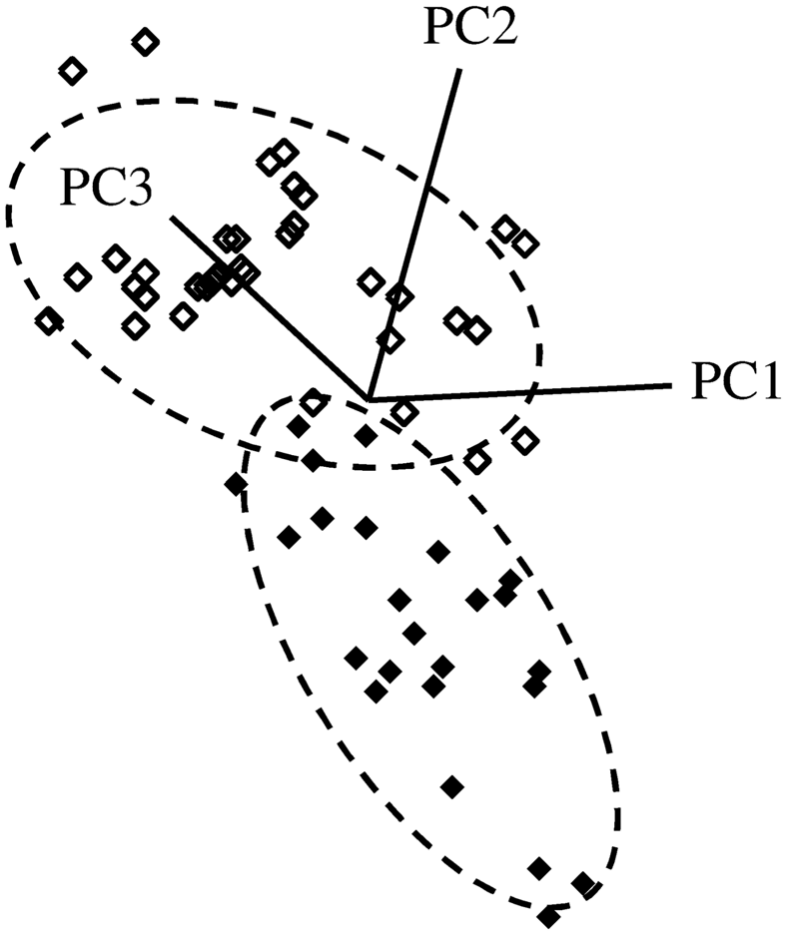
**SIMCA 3D class projection plot for spectral data, transformed using SNV and second derivative**. Data collected from the Excalibur 3500GX (benchtop) unit equipped with triple-bounce ATR accessory. Closed diamonds—resistant trees; open diamonds—susceptible trees. Dashed lines indicate the 95% confidence interval for each group.

**Figure 5:**
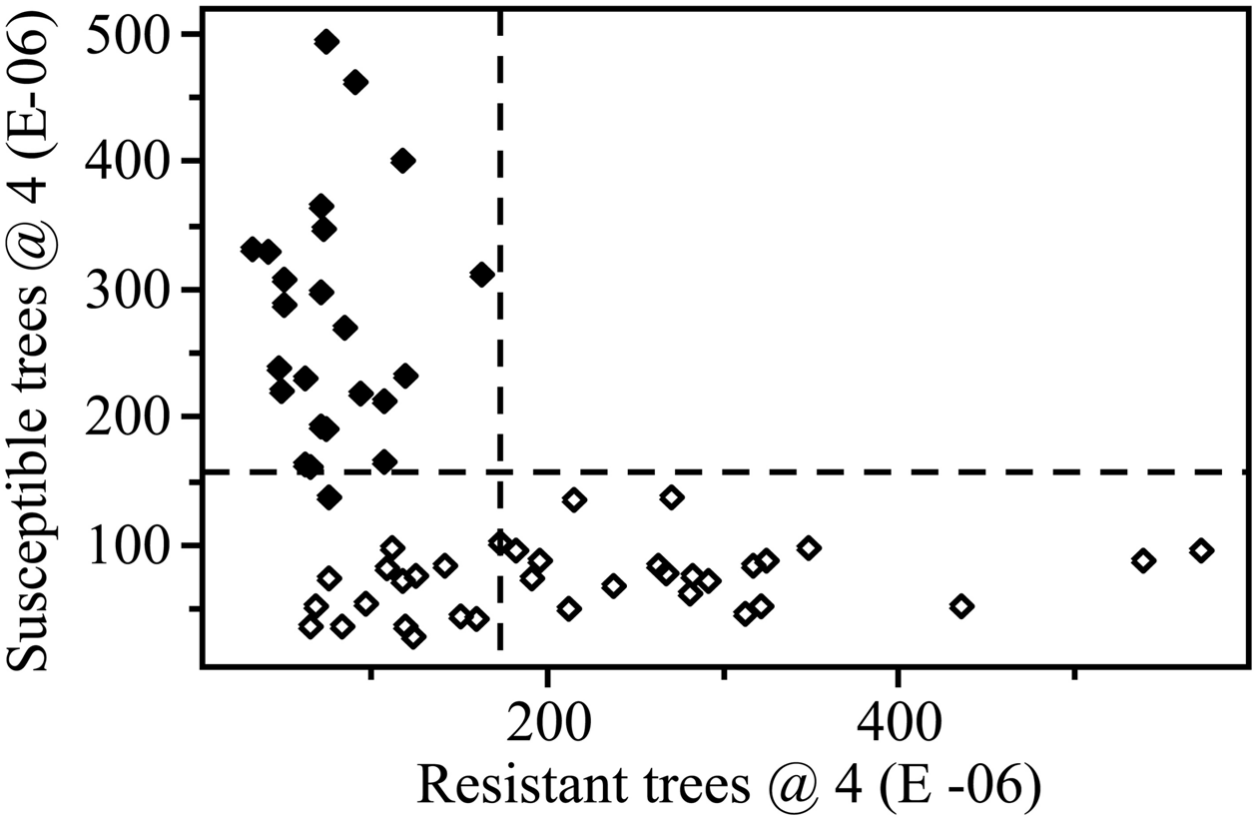
**SIMCA Coomans plot with 4 factors (dashed lines indicate critical sample residual thresholds) based on transformed (SNV and second derivative) data**. Data from the Excalibur 3500GX (benchtop) unit equipped with triple-bounce ATR accessory. Closed diamonds—resistant trees; open diamonds—susceptible trees. This plot shows the relative, dimension-free distance of a sample from a given class, resistant (x-axis) or susceptible (y-axis), based on the 4 factor SIMCA analysis.

A 4-factor SIMCA identified two spectral regions that were most important for reliably discriminating between resistant and susceptible trees, regardless of instrument used. This included spectra from ~1250 to 1350 cm^−1^ and 1700 to 1800 cm^−1^, which corresponded primarily to carbonyl (C=O) group stretching vibrations. Using data collected from the benchtop system (with outliers removed), 100% of extracts from resistant trees (*n* = 24) and 100% of extracts from susceptible trees (*n* = 36) were correctly classified, with an interclass distance of 2.4 (the larger the interclass distance, the less likely samples will be classified as both resistant and susceptible by the SIMCA model) (Figures 4, 5). For data collected from the portable unit (with outliers removed), 100% of extracts from resistant trees (*n* = 25) and 97% of extracts from susceptible trees (*n* = 31) were correctly classified, with an interclass distance of 2.3 (Figures S1, S2).

### PLSR analysis

**Figure 6:**
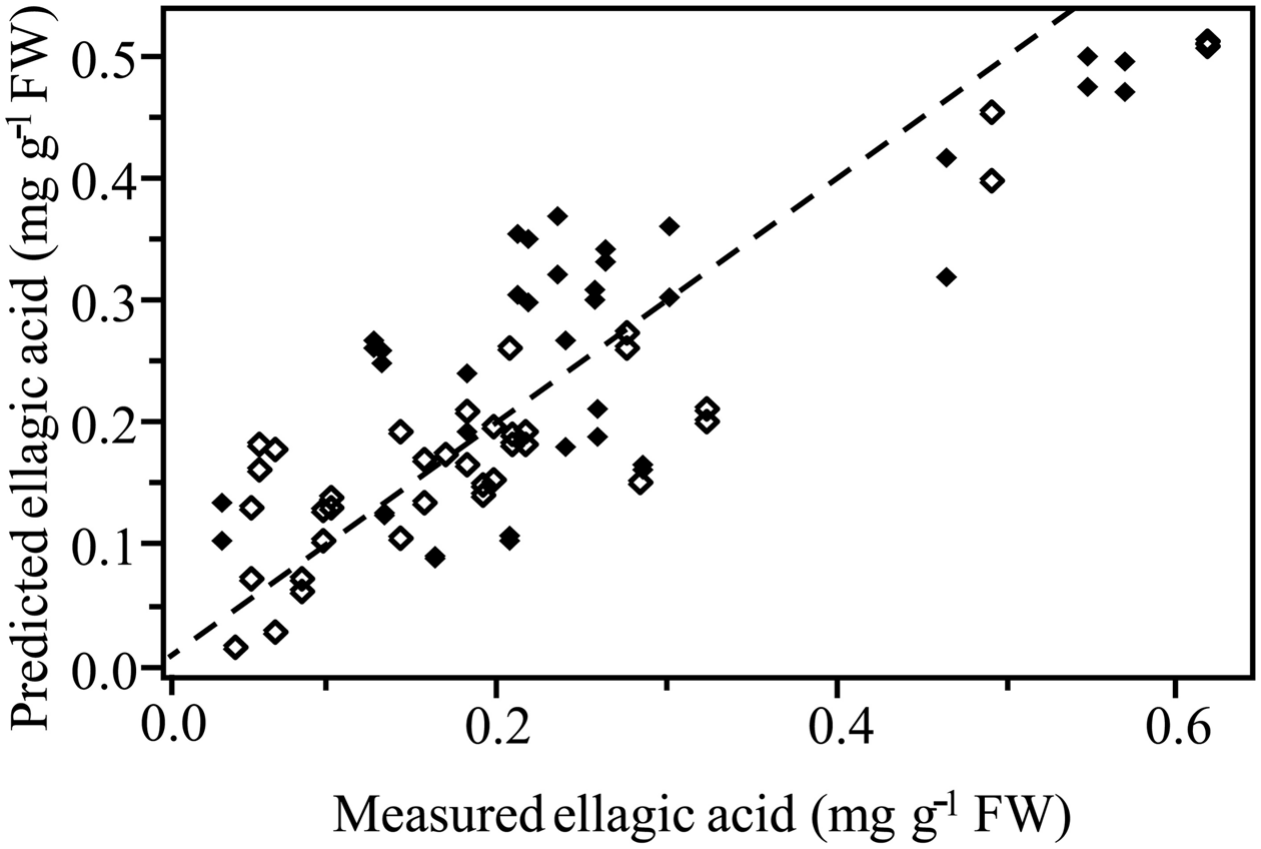
**PLSR correlation plot showing the relationship between the concentration of the phenolic biomarker of resistance, ellagic acid, determined by HPLC analysis, and the predicted concentration of ellagic acid based on FT-IR spectra**. Spectra collected from the Excalibur 3500GX (benchtop) unit equipped with triple-bounce ATR accessory. Spectral data were normalized with divide by sample 2-norm transformation. Closed diamonds—resistant trees; open diamonds—susceptible trees. Statistical analysis reported in Table 2.

**Figure 7:**
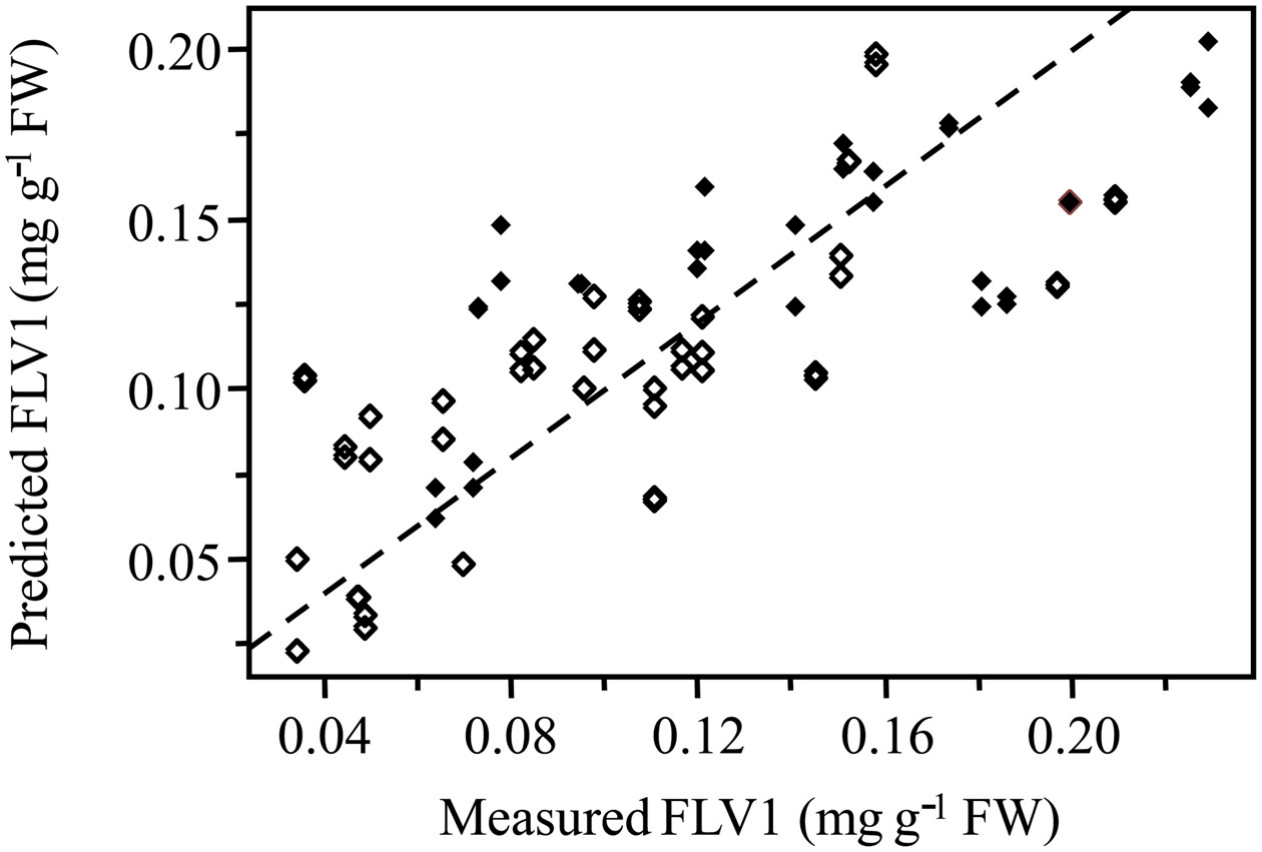
**PLSR correlation plot showing the relationship between the concentration of the phenolic biomarker of resistance, FLV1, in ellagic acid equivalents (mg g^−1^ FW), determined by HPLC analysis, and the predicted concentration of FLV1 based on FT-IR spectra**. Spectra collected from the Excalibur 3500GX (benchtop) unit equipped with triple-bounce ATR accessory. Spectral data were normalized with divide by sample 2-norm transformation. Closed diamonds—resistant trees; open diamonds—susceptible trees. Statistical analysis reported in Table 2.

**Figure 8:**
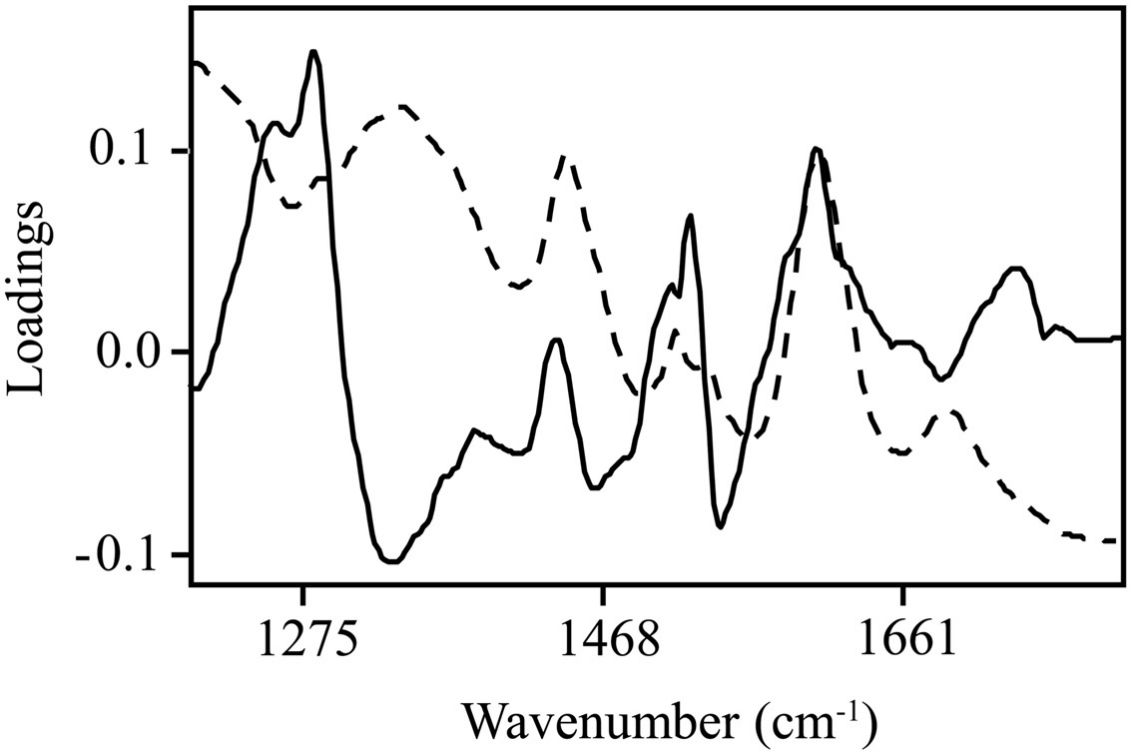
**Ellagic acid PLSR loadings plot with divide by sample 2-norm transformed data for the 4th factor (solid line), the main factor of discrimination between resistant and susceptible trees, with raw spectra (absorbance) overlaid (dashed line)**. Data collected from the Excalibur 3500GX (benchtop) unit equipped with triple-bounce ATR accessory. High loading values, either positive or negative, indicate informative spectra.

**Figure 9:**
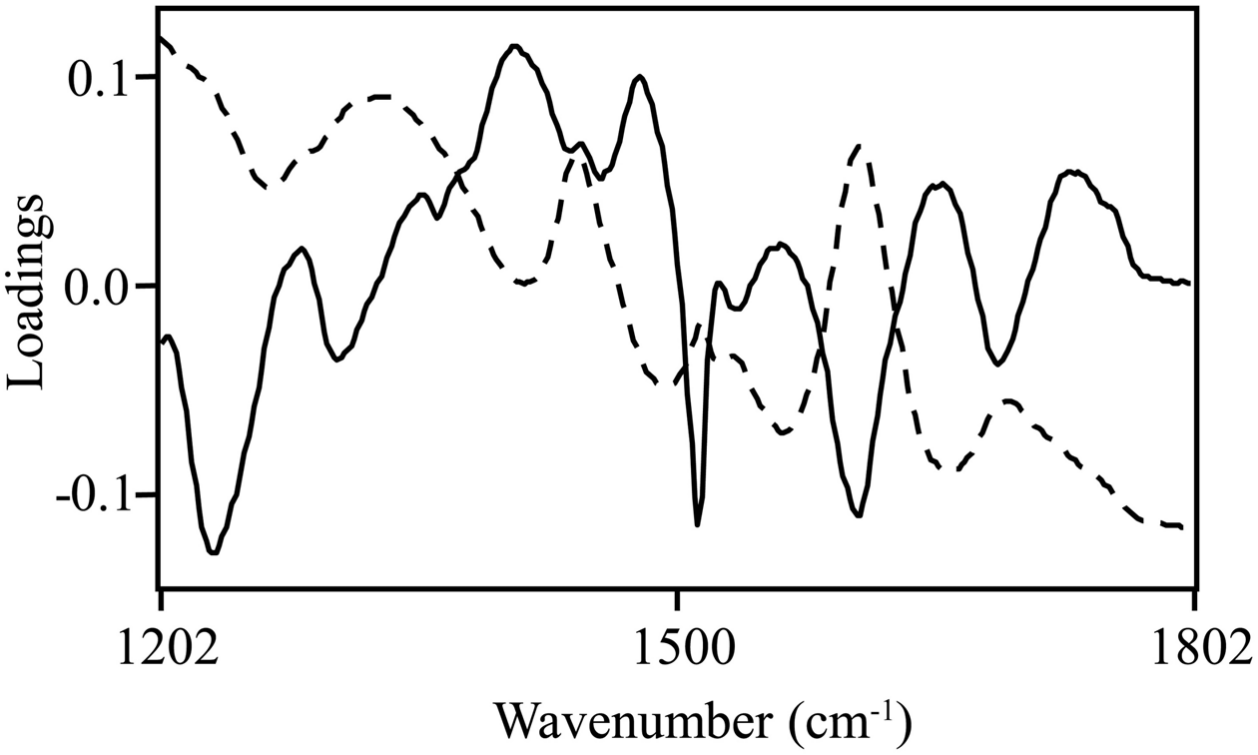
**FLV1 PLSR loadings plot with divide by sample 2-norm data for the 3rd factor (solid line), the main factor of discrimination between resistant and susceptible trees with raw spectra (absorbance) overlaid (dashed line)**. Data collected from the Excalibur 3500GX (benchtop) unit equipped with triple-bounce ATR accessory. High loading values, either positive or negative, indicate informative spectra.

Normalized (divide by sample 2-norm) spectra between 1202–1802 cm^−1^ (benchtop unit) and 1200–1801 cm^−1^ (portable unit) could be used to predict the concentration of two putative phenolic biomarkers of resistance, ellagic acid and FLV1, independently (Table 2). For ellagic acid, a 4-factor PLSR analysis explained >99.9% of the variation in the concentration of ellagic acid, regardless of instrument used, with a strong positive correlation (r_benchtop_ = 0.84; r_portable_ = 0.75) between the predicted and measured concentrations (Figure 6, Figure S3). The standard error of cross-validation (SECV), an approximation of the anticipated error when independent samples are predicted using the model, for ellagic acid was 0.08–0.09%. A 3-factor PLSR analysis explained >99.9% of the variation in the concentration of FLV1, regardless of instrument used, with a strong positive correlation (r_benchtop_ = 0.78; r_portable_ = 0.84) between measured and predicted concentrations and a SECV of 0.03% (Figure 7, Figure S4). Loadings plots for factor 4 (ellagic acid) and factor 3 (FLV1) overlaid with preprocessed spectral data indicate areas of the spectrum which correspond with high loading values (either positive or negative) for ellagic acid (Figure 8, Figure S5) and FLV1 (Figure 9, Figure S6). Areas of the spectrum overlapped with high loading values are likely important for predicting the concentration of each biomarker.

**Table 2:** **Results of PLSR analysis for ellagic acid and FLV1**.

**Compound**	**Range (mg g^−1^ FW)**	**Instrument**	**# Factors**	**% variance explained**	r^*^_val_	SECV^**^ (mg g^−1^FW)	% N Removed^***^
Ellagic acid	0.04–0.62	Benchtop	4	99.94	0.84	0.08	15
		Portable	4	99.94	0.75	0.09	13
FLV1^****^	0.03–0.47	Benchtop	3	99.91	0.78	0.03	15
		Portable	3	99.92	0.84	0.03	22

* *Correlation coefficient of cross-validation—correlation coefficient describing the direction and strength of the relationship between cross-validated predicted and actual concentrations*.

** *Standard error of cross-validation—standard error of cross-validated predicted concentrations*.

*** *N = 93*.

**** *In ellagic acid equivalents*.

## Discussion

For the first time, we demonstrate that chemical fingerprinting, based on FT-IR spectroscopy of phloem extracts combined with chemometric analysis, can be used to predict resistance in a natural population of CLOs prior to infection by an important invasive pathogen, *P. ramorum*. Chemical fingerprints were also used to predict the concentration of two putative phenolic biomarkers of CLO resistance, ellagic acid (McPherson et al., 2014) and FLV1 (Conrad et al., unpublished). Variability in spectral intensities was observed within resistant and susceptible trees; however, resolution of overlapping peaks by normalization and transformation, along with outlier trimming, made it possible to develop strong predictive models. Spectral bands corresponding to carbonyl group vibrations were consistently important for distinguishing between resistant and susceptible CLO, regardless of instrument sensitivity. Instrument sensitivity was dependent on the specific configuration of the ATR accessory, with the five-bounce ATR accessory (portable unit) more likely to detect subtle differences between groups than the triple-bounce ATR accessory (benchtop unit) (Agilent Technologies, 2013).

Two spectral ranges, corresponding primarily to carbonyl group vibrations, had the greatest discriminating power between resistant and susceptible trees in 3-dimensional space using a 4-factor SIMCA. While spectral bands with the greatest discriminating power differed slightly depending on the instrument used, in general, bands previously associated with plant specialized metabolites were most important. Band 1305 cm^−1^ was previously found in free quercetin (Torreggiani et al., 2005). Quercetin may be important for plant defense because under certain conditions it scavenges free radicals and chelates metal ions (Torreggiani et al., 2005). Furthermore, quercetin inhibits the growth of *Phytophthora megasperma* Drechsler *in vitro*, a pathogen of olive roots and many other woody species (Báidez et al., 2006). Two bands at 1735 cm^−1^ (Bovi Mitre et al., 2004) and 1772 cm^−1^ (Genta et al., 2010) were previously associated with lactones. While we do not know exactly which compounds in our extracts are responsible for these two bands, we do know that ellagic acid is a dilactone (Ascacio-Valdes et al., 2011) with absorbance in the same range that we used for the SIMCA analysis (Figure S7). Moreover, this corroborates previous studies, which examined the relationship between CLO phenolics and defense and/or resistance to *P. ramorum*. Ockels et al. (2007) found that ellagic acid was associated with CLO defense, and Nagle et al. (2011) found that trees more resistant to *P*. *ramorum* had higher levels of ellagic acid in their phloem tissue compared to more susceptible CLO. McPherson et al. (2014) identified ellagic acid as one of four putative phenolic biomarkers of resistance in asymptomatic tissue of already infected trees, and also found that ellagic acid inhibited the growth of *P. ramorum* at *in planta*-relevant concentrations *in vitro*.

In addition to using FT-IR spectra to discriminate between resistant and susceptible CLO, PLSR was used to predict the concentration of two putative phenolic biomarkers of resistance, ellagic acid (McPherson et al., 2014) and FLV1 (Conrad et al., unpublished). Predicted concentrations of each phenolic biomarker were strongly positively correlated with measured concentrations of each compound, regardless of instrument used, confirming that FT-IR spectroscopy can be used to identify and/or quantify phytochemical features associated with resistant trees. Bands associated with aromatic ring (C=C) and carbonyl (C=O) group vibrations had the highest loading values. Some of the bands identified as being important, based on correlation spectrum plots (data not shown), were previously found to be associated with oak tannin (Gust, 1991), C=O stretching associated with elm defense (Martín et al., 2005), and phenols and C-C bending in gallic acid (Mohammed-Ziegler and Billes, 2002). The potential association of one of these bands with gallic acid is of particular interest, since gallic acid was found at higher concentrations in *P. ramorum* infected phloem tissue and has been shown to inhibit the growth of multiple *Phytophthora* species *in vitro*, including *P. ramorum* (Ockels et al., 2007).

Taken together these results suggest that FT-IR spectroscopy is a viable approach for chemically fingerprinting methanol extracts from CLO phloem tissue. By performing chemometric analysis on data collected from FT-IR spectroscopy, we were able to (1) discriminate between CLO resistant and susceptible to *P. ramorum* prior to infection with the pathogen and (2) estimate the concentration of two putative constitutive phenolic biomarkers of resistance. In the future, these models can be used to predict whether or not an uninfected CLO will be resistant to *P. ramorum*, though they may need to be refined (by incorporating data from additional CLO), depending on the accuracy required in future predictions.

Knowledge of resistant (or susceptible) CLO in the landscape may be useful for homeowners, extension agents, or forest managers interested in protecting high-value trees with chemical treatments, protecting stands with high levels of resistance from development and fire, or for the development of sudden oak death management and risk assessment plans. In some areas where many resistant trees are present, the best form of management may be no intervention (allowing naturally resistant trees to replenish the seed bank), or may be limited only to the removal of hazardous trees. Furthermore, the approach detailed in this study may be appropriate for use in other forest pathogen and pest systems where the main objective is to identify resistant germplasm.

### Conflict of interest statement

The authors declare that the research was conducted in the absence of any commercial or financial relationships that could be construed as a potential conflict of interest.
