# Corrigendum: Identification of *Quercus agrifolia* (coast live oak) resistant to the invasive pathogen *Phytophthora ramorum* in native stands using Fourier-transform infrared (FT-IR) spectroscopy

**DOI:** 10.3389/fpls.2017.01849

**Published:** 2017-10-20

**Authors:** Anna O. Conrad, Luis E. Rodriguez-Saona, Brice A. McPherson, David L. Wood, Pierluigi Bonello

**Affiliations:** ^1^Department of Plant Pathology, The Ohio State University, Columbus, OH, United States; ^2^Department of Food Science and Technology, The Ohio State University, Columbus, OH, United States; ^3^Department of Environmental Science, Policy and Management, University of California, Berkeley, CA, United States

**Keywords:** coast live oak, resistance, sudden oak death, infrared spectroscopy, predictive modeling

In the original article, there was an error. Units for standard error of cross-validation (SECV) were incorrectly listed in the text on page 5 as %, but should have been listed as mg g^−1^ FW, as is stated in **Table 2**.

A correction has been made to Results, PLSR Analysis, paragraph one, on page 5:

Normalized (divide by sample 2-norm) spectra between 1,202–1,802 cm^−1^ (benchtop unit) and 1,200–1,801 cm^−1^ (portable unit) could be used to predict the concentration of two putative phenolic biomarkers of resistance, ellagic acid and FLV1, independently **(Table 2)**. For ellagic acid, a 4-factor PLSR analysis explained >99.9% of the variation in the concentration of ellagic acid, regardless of instrument used, with a strong positive correlation (*r*_benchtop_ = 0.84; *r*_portable_ = 0.75) between the predicted and measured concentrations (**Figure 6**, Figure S3). The standard error of cross-validation (SECV), an approximation of the anticipated error when independent samples are predicted using the model, for ellagic acid was 0.08–0.09 mg g^−1^ FW. A 3-factor PLSR analysis explained >99.9% of the variation in the concentration of FLV1, regardless of instrument used, with a strong positive correlation (*r*_benchtop_ = 0.78; *r*_portable_ = 0.84) between measured and predicted concentrations and a SECV of 0.03 mg g^−1^ FW (**Figure 7**, Figure S4). Loadings plots for factor 4 (ellagic acid) and factor 3 (FLV1) overlaid with preprocessed spectral data indicate areas of the spectrum which correspond with high loading values (either positive or negative) for ellagic acid (**Figure 8**, Figure S5) and FLV1 (**Figure 9**, Figure S6). Areas of the spectrum overlapped with high loading values are likely important for predicting the concentration of each biomarker.

The authors apologize for this error and state that this does not change the scientific conclusions of the article in any way.

## Conflict of interest statement

The authors declare that the research was conducted in the absence of any commercial or financial relationships that could be construed as a potential conflict of interest.

