# Supplementary material for: Identification of Quercus agrifolia (coast live oak) resistant to the invasive pathogen Phytophthora ramorum in native stands using Fourier-transform infrared (FT-IR) spectroscopy
Source: Front Plant Sci. 2014 Oct 14;5:521. doi: 10.3389/fpls.2014.00521 (PMC4196480; doi:10.3389/fpls.2014.00521)
Supplement: Supplementary file 1 [file DataSheet1.DOCX]

***Supplementary Material***

**Identification of *Quercus agrifolia* (coast live oak) resistant to the invasive pathogen *Phytophthora ramorum* in native stands using Fourier-transform infrared (FT-IR) spectroscopy**

**Anna O. Conrad^1,^*, Luis E. Rodriguez-Saona^2^, Brice A. McPherson^3^, David L. Wood^3^, Pierluigi Bonello^1^**

^1^Department of Plant Pathology, The Ohio State University, Columbus, OH, USA

^2^Department of Food Science and Technology, The Ohio State University, Columbus, OH, USA

^3^Department of Environmental Science, Policy, and Management, University of California, Berkeley, CA, USA

*** Correspondence:** Anna O. Conrad, Department of Plant Pathology, The Ohio State University, 201 Kottman Hall, 2021 Coffey Road, Columbus, OH, 43210, USA.

conrad.245@osu.edu

1. **Supplementary Figures**


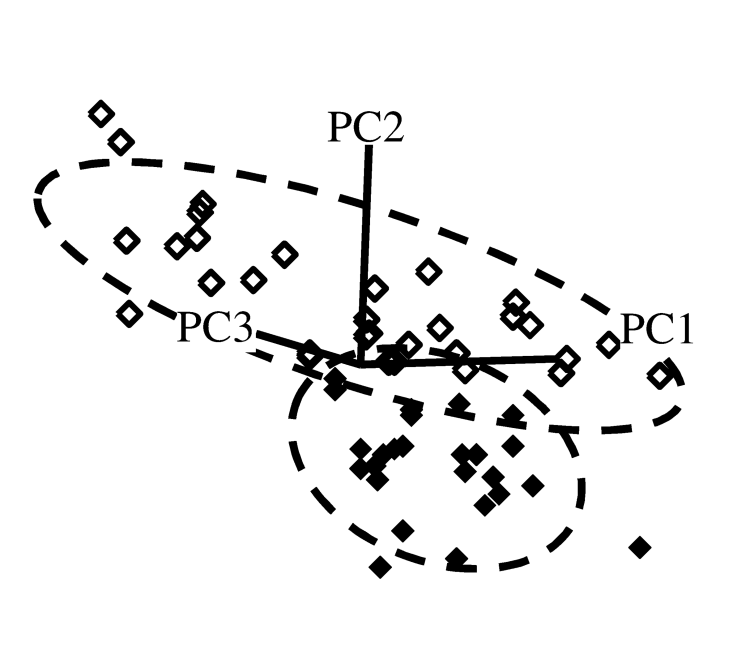


**Supplemental Figure 1 SIMCA 3D class projection plot for spectral data, transformed using SNV and second derivative.** Data collected from the Cary 630 (portable) FT-IR unit equipped with 5-bounce ATR accessory. Closed diamonds—resistant trees; open diamonds—susceptible trees. Dashed lines indicate the 95% confidence interval for each group.

**
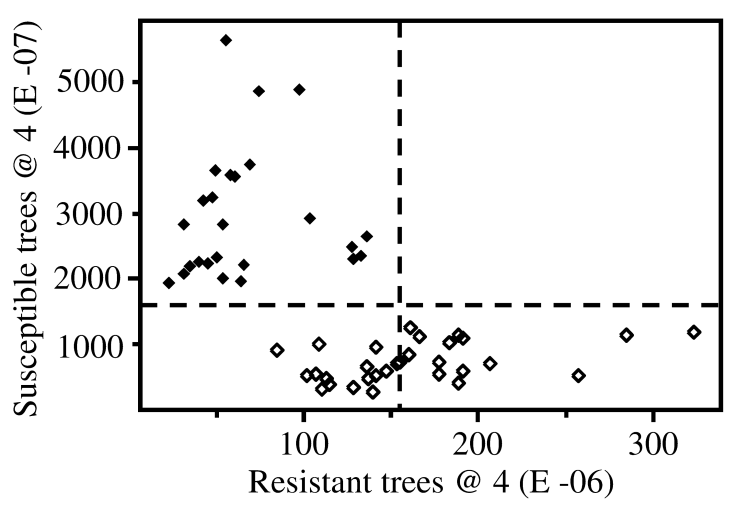
**

**Supplemental Figure 2 SIMCA Coomans plot with 4 factors (dashed lines indicate critical sample residual thresholds) based on transformed (SNV and second derivative) data.** Data collected from the Cary 630 (portable) unit equipped with 5-bounce ATR accessory. Closed diamonds—resistant trees; open diamonds—susceptible trees. This plot shows the relative dimension-free distance of a sample from a given class, resistant (x-axis) or susceptible (y-axis), based on the 4 factor SIMCA analysis.

**
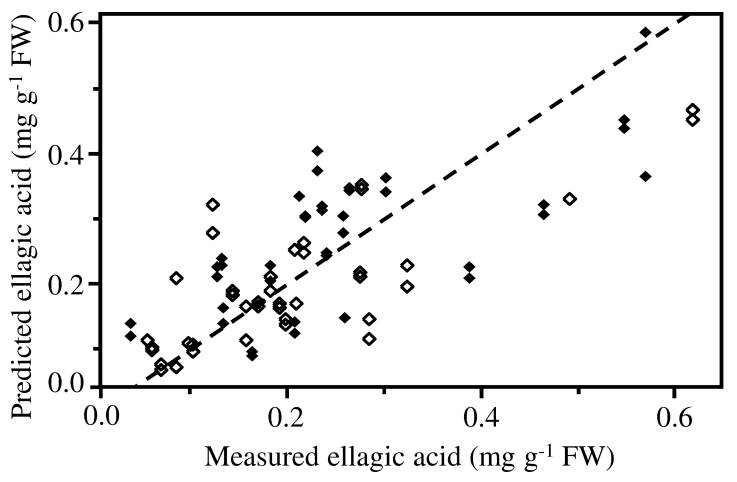
**

**Supplemental Figure 3 PLSR correlation plot showing the relationship between the concentration of the phenolic biomarker of resistance, ellagic acid, determined by HPLC analysis, and the predicted concentration of ellagic acid based on FT-IR spectra**. Data collected from the Cary 630 (portable) unit equipped with 5-bounce ATR accessory. Spectral data were normalized with divide by sample 2-norm transformation. Closed diamonds—resistant trees; open diamonds—susceptible trees. Statistical analysis reported in Table 2.


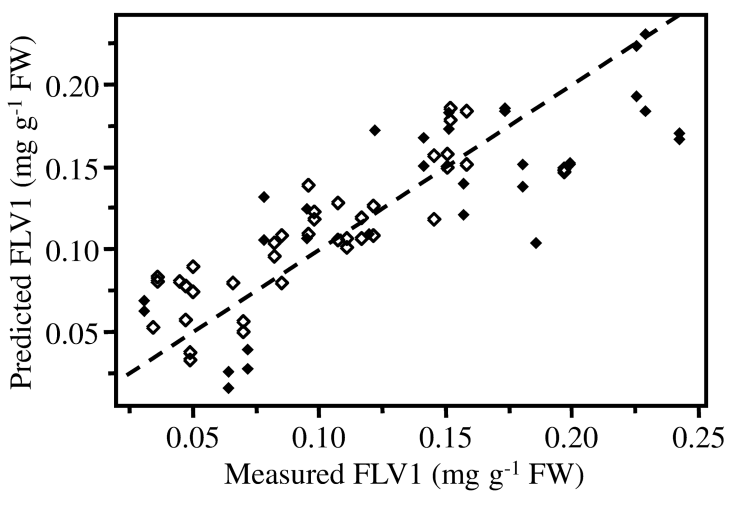


**Supplemental Figure 4 PLSR correlation plot showing the relationship between the concentration of the phenolic biomarker of resistance, FLV1, in ellagic acid equivalents (mg g^-1^ FW), determined by HPLC analysis, and the predicted concentration of FLV1 based on FT-IR spectra.** Spectra collected from the Cary 630 (portable) unit equipped with 5-bounce ATR accessory. Spectral data were normalized with divide by sample 2-norm transformation. Closed diamonds—resistant trees; open diamonds—susceptible trees. Statistical analysis reported in Table 2.


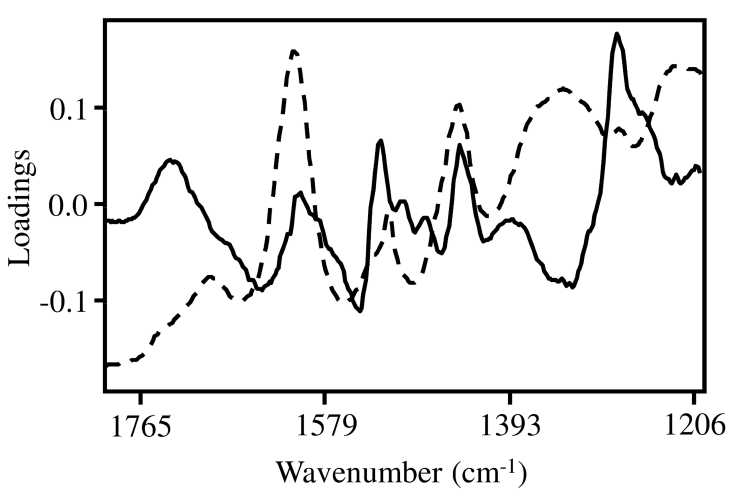


**Supplemental Figure 5 Ellagic acid PLSR loadings plot with divide by sample 2-norm transformed data for the 4^th^ factor (solid line), the main factor of discrimination between resistant and susceptible trees, with raw spectra (absorbance) overlaid (dashed line) for data.** Data collected from the Cary 630 (portable) unit equipped with 5-bounce ATR accessory. High loading values, either positive or negative, indicate informative spectra.


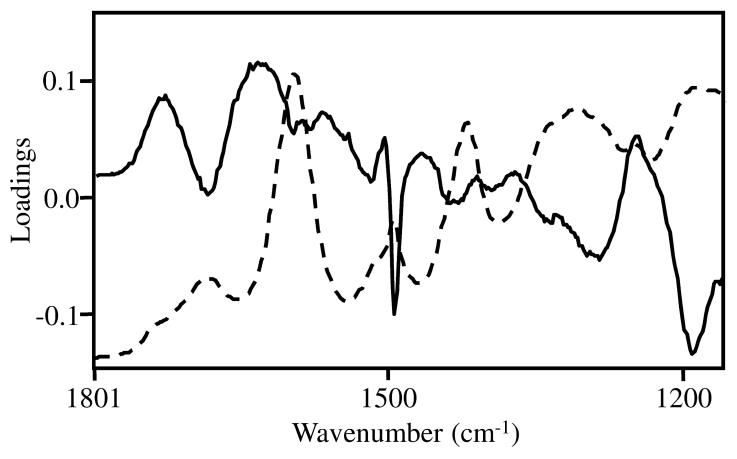


**Supplemental Figure 6 FLV1 PLSR loadings plot with divide by sample 2-norm data for the 3^rd^ factor (solid line), the main factor of discrimination between resistant and susceptible trees with raw spectra (absorbance) overlaid (dashed line) for data.** Data collected from the Cary 630 (portable) unit equipped with 5-bounce ATR accessory. High loading values, either positive or negative, indicate informative spectra.

**
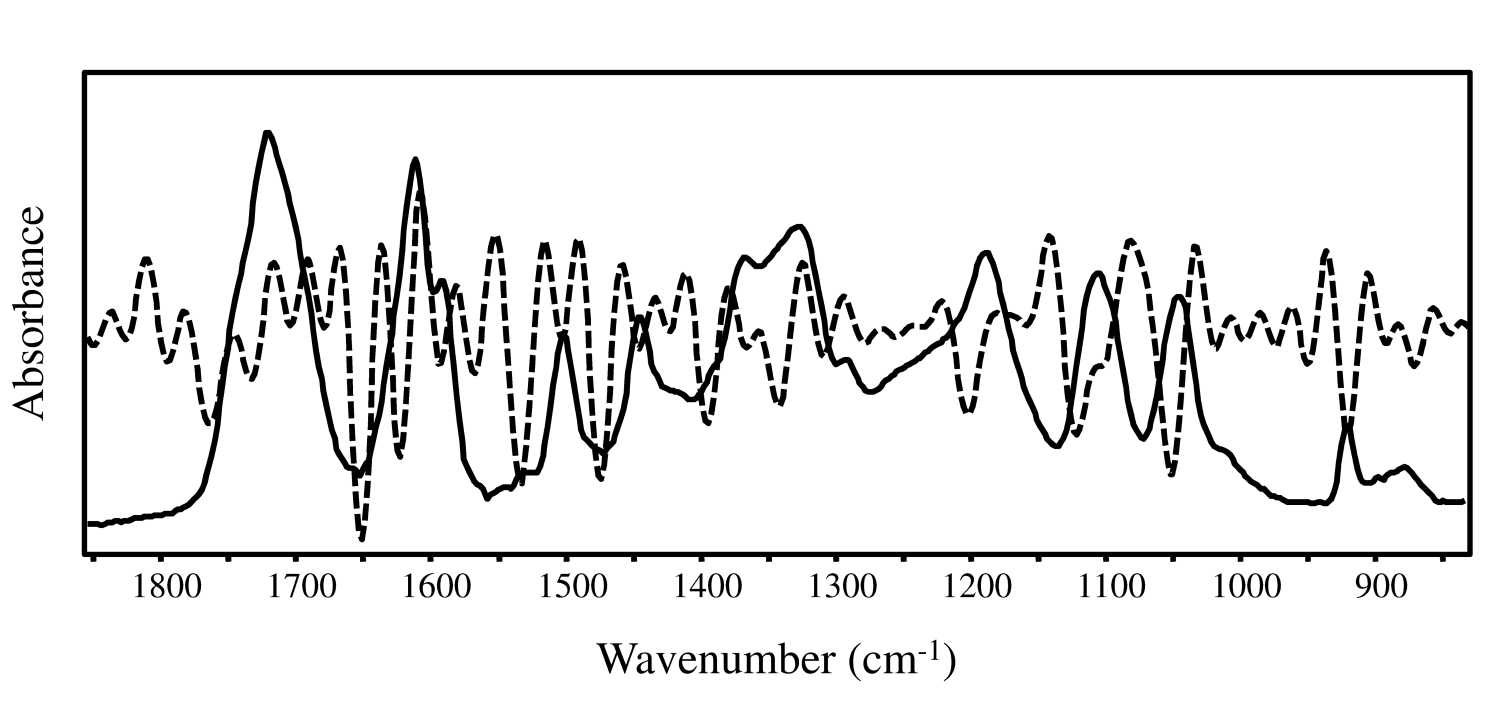
**

**Supplemental Figure 7 Ellagic acid standard FT-IR spectra.** Solid line—raw data; dashed line—second derivative data.
